# Characterization and Antimicrobial Activity of Erbium(III) Complexes of C-3 Substituted 2-hydroxy-1,4-naphthalenedione-1-oxime Derivatives

**DOI:** 10.1155/MBD.2001.159

**Published:** 2001

**Authors:** S. B. Jagtap, N. N. Patil, B. P. Kapadnis, B. A. Kulkarni

**Affiliations:** 1 Department of Chemistry University of Pune Pune Pune-411 007 India; 2 Department of Microbiology University of Pune Pune Pune- 411 007 India

## Abstract

Erbium(III) complexes of 2-hydroxy-l,4-naphthalenedione-1-oxime and its C-3 substituted derivatives are synthesized and characterized by elemental analysis, thermogravimetric analysis, infrared spectroscopy, magnetic susceptibility measurements 2-hydroxy-1,4-naphthalenedione-1-oxime derivatives are analysed using ^1^H and ^13^C NMR spectroscopy. The molecular composition of the synthesized complexes is found to be [ML_3_(H_2_O)_2_]. The antimicrobial activity of these complexes is determined by well diffusion method against the target microorganisms- *Staphylococcus aureus, Xanthomonas campestris, Pseudomonas aeruginosa, Candida albicans * and *Aspergillus niger*. The antimicrobial activities of 2- hydroxy-1,4-naphthalenedione-1-oximes and their complexes are compared. It is observed that 2-hydroxy-1,4-naphthalenedione-l-oximes exhibit higher antifungal activity as compared to antibacterial activity. These activities are reduced upon complexation of these oximes with Erbium.


### CHARACTERIZATION AND ANTIMICROBIAL ACTIVITY OF ERBIUM(III) COMPLEXES OF C-3 SUBSTITUTED 2-HYDROXY-1,4-NAPHTHALENEDIONE-1-OXIME DERIVATIVES

S. B. Jagtap[1], N.N.Patil [2], B.P.Kapadnis*[2] and B. A. Kulkarni[1]

[1]Department of Chemistry, [2]Department of Microbiology,
University of Pune, Pune - 411 007, India.
* Corresponding Author Fax: + 91 20 5650087 E-mail: bpkap@hotmail.com

### ABSTRACT

Erbium(III) complexes of 2-hydroxy-1,4-naphthalenedione-1-oxime and its C-3 substituted derivatives are synthesized and characterized by elemental analysis, thermogravimetric analysis, infrared spectroscopy, magnetic susceptibility measurements. 2-hydroxy-1,4-naphthalenedione-1-oxime derivatives are analysed using $^1H$ and $^{13}C$ NMR spectroscopy. The molecular composition of the synthesized complexes is found to be $[ML_3(H_2O)_2]$. The antimicrobial activity of these complexes is determined by well diffusion method against the target microorganisms- *Staphylococcus aureus, Xanthomonas campestris, Pseudomonas aeruginosa, Candida albicans* and *Aspergillus niger.* The antimicrobial activities of 2-hydroxy-1,4-naphthalenedione-1-oximes and their complexes are compared. It is observed that 2-hydroxy-1,4-naphthalenedione-1-oximes exhibit higher antifungal activity as compared to antibacterial activity. These activities are reduced upon complexation of these oximes with Erbium.

### INTRODUCTION

The utilization of the lanthanides and their complexes in biological and biochemical studies has been reviewed by Williams[1]. The application for localization of tumors using appropriate lanthanide complexes was studied by Hider *et al.*[2] and Lauffer[3]. Yam *et al.* have reported that lanthanide complexes play a key role in various dignostic areas[4]. Gaikwad[5] and Dandawate[6] have studied the complexes of some lanthanide metals with hydroxy-naphthoquinone-oximes. In continuation of studies on lanthanide complexes as antimicrobial agents[5-7], we are reporting antimicrobial activity of Erbium(III) complexes by well diffusion assay method. General structure of the ligating system(Fig.1) is as reported earlier[8].

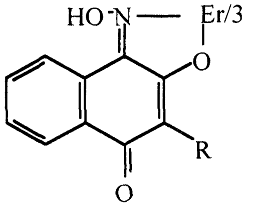

| R | Ligand |
|---|---|
| H | 2-hydroxy-1,4-naphthoquinone-1-oxime ($HL_1$) : (I) |
| $CH_3$ | 2-hydroxy-3-methyl-1,4-naphthoquinone-1-oxime ($HL_2$): (II) |
| Cl | 2-hydroxy-3-chloro-1,4-naphthoquinone-1-oxime ($HL_3$): (III) |
| Br | 2-hydroxy-3-bromo-1,4-naphthoquinone-1-oxime ($HL_4$): (IV) |
| I | 2-hydroxy-3-iodo-1,4-naphthoquinone-1-oxime ($HL_5$): (V) |

**Figure 1**: General structure of ligating system

### MATERIALS AND METHODS

### Synthesis of ligands

All the chemicals and solvents used were of analytical grade. 2-hydroxy-1,4-naphthalenedione (lawsone), 2,3-dichloro-1,4-naphthalenedione(dichlone) and 2-methyl-1,4-naphthalenedione (menadione) were purchased from Fluka (Germany). 2-hydroxy-3-methyl-1,4-naphthalenedione (phthiocol) was prepared from menadione by Fieser's method[9]. The 2-hydroxy-3-chloro-1,4-naphthalenedione was synthesized from dichlone. All the ligands (2-hydroxy-1,4-naphthalenedione-1-oxime derivatives) were prepared by the method reported in earlier paper[8]. They were recrystallized using methanol and melting points were recorded.

### Synthesis of complexes

To a hot solution of 3 mmol of oxime derivative [0.568 g of (I), 0.60 g of (II), 0.671 g of (III), 0.804 g of (IV) and 0.945 g of (V)] in 25 mL of ethanol, an aqueous solution of 1 mmol of erbium trichloride hexahydrate (0.381 g ) was added. The pH of the mixture was kept around 6 using aqueous ammonia (1:20 v/v). It was refluxed for 3 h and then cooled overnight. The precipitate was filtered off, washed with water, followed by hot methanol and dried in vacuum over fused $CaCl_2$ at ambient temperature. The solubility of these complexes was tested in $H_20$, $CH_3OH$, $CH_3CN$, DMF, DMSO and inert solvents.

The elemental analysis was carried out using a Hosli-Holland C, H Analyzer. The magnetic studies were carried out at room temperature by the Faraday technique using mercury(II) tetrathiocyanatocobaltate as calibrant. The Infrared spectra were recorded in nujol mulls on a Perkin-Elmer FTIR spectrophotometer (Model 1600,4000-450cm$^{-1}$). $^{1}H$ NMR and $^{13}C$ NMR spectra were recorded on Varian Mercury-300 NMR spectrophotometer.

**Media**

The dehydrated plate count medium (g/100 ml distilled water : glucose 0.1, yeast extract 0.25, tryptone 0.5) and Sabouraud's dextrose agar ( g/100 ml distilled water : glucose 4, peptone) purchased from Hi- Media Laboratories, India were used respectively for antibacterial and antifungal activity.

**Microorganisms and their maintenance**

The target microorganisms included *Staphylococcus aureus* NCIM2079, *Xanthomonas campestris* NCIM 2954, *Pseudomonas aeruginosa* NCIM2036, *Candida albicans* NCIM3471 *and Aspergillus niger* NCIM 545. These were obtained from NCIM, NCL, India. These strains were selected because they are routinely used in testing of disinfectants[10]. The stock cultures of these microorganisms were maintained at -20$^{0}$C in 15% glycerol [11]. The inoculum was prepared from stock cultures by streaking onto the plate count agar for bacteria and on Sabouraud's dextrose agar for fungi. After an overnight incubation single colony was used to inoculate sterile liquid media. The 5ml broth was dispensed in test tubes and sterilized in the autoclave at 121$^{0}$C for 15 min. The broths were then inoculated with respective cultures and incubated on an orbital shaker (150 rpm) overnight at 30$^{0}$C $A_{540}$ of bacterial cultures and *Candida albicans.* was adjusted to 0.12 and 0.20 respectively. This corresponds to $10^{6}$-$10^{7}$ colony forming unit (cfu/ml). The spore inoculum of *A. niger* containing $10^{6}$ spores per ml was used.

**Determination of Minimum cidal concentration(MCC) by the well diffusion assay method**

The solutions of ligands and complexes prepared in DMSO [12] were diluted in DMSO and added to tubes containing 3 mL liquid medium and inoculated with 30 µL of the cultures. Incubation was done for 18 h at 37$^{0}$C. The minimum cidal concentration was then found out. This concentration was used for well diffusion assay method[13].

**Well diffusion assay method**

8 mm diameter wells were made in the set agar in petri dish, previously spread with 50 µL inoculum of target cultures. The wells were loaded with 30 µL of test compounds. The plates were pre-incubated for 2 h at 4$^{0}$C and then incubated for 18 h at 37$^{0}$C. For *A. niger* the incubation was done for 72 h at room temperature. The zone of inhibition was then measured. The control experiments were performed using equivalent volume of the solvent itself loaded into the wells. All the values of inhibition zone are average of three replicate experiments. The standard deviation of all values was within 5% of arithmetic mean.

### RESULTS AND DISCUSSION

The reaction involving Er(III) and 2-hydroxy-1,4-naphthalenedione-1-oximes in ethanolic medium with 1:3 metal-ligand(M:L) ratio resulted in the complexes having molecular composition as $ErL_3.(H_2O)_2$ (where L - anion of the corresponding oxime derivatives) which is supported by elemental analysis. All the oxime derivatives are soluble in water, methanol, DMF, DMSO and acetonitrile, while their complexes are soluble in DMF, DMSO, methanol and acetonitrile and are insoluble in inert solvents like n-hexane, benzene, 1,4-dioxane etc (Table 1). The elemental analysis of ligands is already reported [8].

**Table 1**. Elemental analysis and physical properties of metal complexes

| Complex. | Colour | Decom. Temp. (° C) | Yield (%) | Elemental Analysis found (calc.) % C | % H | % N | % M | $\mu_{eff}$*. (BM) |
|---|---|---|---|---|---|---|---|---|
| $Er_1$ | Greenish Canary yellow | 295 | 71.56 | 47.06 (46.93) | 2.76 (2.89) | 5.29 (5.47) | 21.55 (21.78) | 9.11 |
| $Er_2$ | Light orange | 260 | 70.86 | 48.78 (48.94) | 3.54 (3.48) | 5.24 (5.19) | 20.75 (20.65) | 9.40 |
| $Er_3$ | Turmeric yellow | 295 | 71.89 | 40.93 (41.36) | 2.36 (2.19) | 4.65 (4.82) | 19.05 (19.20) | 9.28 |
| $Er_4$ | Dark turmeric yellow | 280 | 73.25 | 36.83 (37.09) | 2.23 (1.97) | 4.17 (4.33) | 17.48 (17.21) | 9.34 |
| $Er_5$ | Dark turmeric yellow | 260 | 68.16 | 31.13 (31.46) | 1.91 (1.67) | 3.42 (3.67) | 14.39 (14.60) | 9.20 |

$Er_1$, Er(III).$(C_{10}H_6O_3N)_3.2H_2O$; $Er_2$, Er (III) $(C_{11}H_8O_3N)_3.2H_2O$; $Er_3$, Er (III)$(C_{10}H_5O_3NCl)_3.2H_2O$; $Er_4$, Er (III) $(C_{10}H_5O_3N.Br)_3.2H_2O$; $Er_5$, Er (III) $(C_{10}H_5O_3N.I)_3.2H_2O$.

*$\mu_{eff}$. is the magnetic moment (BM) at RT.

**a) IR studies**

Selected IR bands of the ligands and complexes are shown in Table 2. A medium broad band at 3100-3600 $cm^{-1}$ exhibited by ligands, is due to the (O-H) vibration of oximino and phenolic hydroxyls functions. Due to overlapping of this stretching frequency with coordinated water molecules, this band is further broadened upon complexation[6]. The redistribution of electron density in the quinonoidal ring is indicated by shifting of (C=O) stretching frequency (1600-1630 $cm^{-1}$) for ligands towards lower frequency region by ~20-30 $cm^{-1}$ is suggestive of complexation. The coordination through the oximino nitrogen reflected by shifting of C=N vibration band (1570-1590 $cm^{-1}$) at lower frequency by ~40-70 $cm^{-1}$ is due to chelation. The quinone absorption is found at 1285-1296 $cm^{-1}$. The shifting of about 10-35 $cm^{-1}$ for (C-O) stretching frequency (1210 $cm^{-1}$) of ligand is indicative of phenolato oxygen as the other coordinating center for oxime derivatives.

**Table 2**. Selected IR bands ($cm^{-1}$) of ligands and erbium complexes.

| Compd+ | ν (O-H) | ν(C=O) | ν (C=N) | Quinone absorption | ν (C-O) | ν (N-O) | ν (C-X*) | ν (Er-O) |
|---|---|---|---|---|---|---|---|---|
| (I) | 3362, 3155 | 1630 | 1576 | 1293 | 1211 | 1050 | -- | -- |
| $Er_1$ | 3267 | 1588 | 1538 | 1287 | 1220 | 1052 | -- | 465 |
| (II) | 3275, 3100 | 1620 | 1587 | 1296 | 1205 | 1052 | -- | -- |
| $Er_2$ | 3314 | 1586 | 1526 | 1294 | 1226 | 1060 | -- | 466 |
| (III) | 3412,3375, 3100 | 1604 | 1577 | 1286 | 1211 | 1050 | 694 | -- |
| $Er_3$ | 3567 | 1580 | 1521 | 1290 | 1224 | 1059 | 691 | 468 |
| (IV) | 3325,3200, 3100 | 1623 | 1589 | 1287 | 1210 | 1055 | 693 | -- |
| $Er_4$ | 3184 | 1580 | 1518 | 1285 | 1223 | 1058 | 689 | 467 |
| (V) | 3325,3187, 3100 | 1620 | 1585 | 1286 | 1224 | 1051 | 692 | -- |
| $Er_5$ | 3440 | 1579 | 1521 | 1279 | 1223 | 1055 | 688 | 474 |

*X is a halogen, +( ) ligand

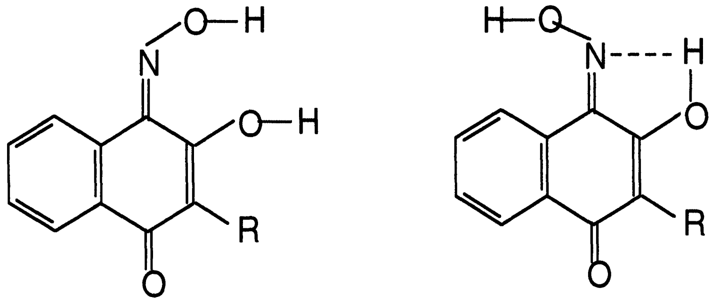

Syn amphi

**Figure 2**. Syn-amphi isomers of 2-hydroxy-1,4-naphthalenedione-1-oxime

The coordination through oximino nitrogen is further confirmed by the increase in the (N-O) stretching frequency (the increase in the 1050 $cm^{-1}$) by 5-20 $cm^{-1}$[14]. Absorption at 688-695 $cm^{-1}$ is assigned to (C-X); this band is shifted to lower region by 3-10 $cm^{-1}$. For complexes, a medium intensity band at 465-475 $cm^{-1}$ is assigned to (Er-O) stretching vibration.

**b) $^{1}H$ and $^{13}C$ NMR studies**

$^{1}H$ and $^{13}C$ NMR data for (I)-(V) is depicted in Table 3and 4 respectively. The syn-amphi isomers of 2-hydroxy-1,4-naphthalenedione-1-oxime derivatives are shown in Fig.2.

It has been reported earlier that $C_2$-hydroxyl signal for lawsone is observed as a broad signal (in $CDCl_3$) at 7.42 ppm indicating a dimeric associations[15]. However, such a dimeric nature was found to be destroyed when it's spectrum was recorded in DMSO-$d_6$; wherein this band appeared at 11.63 ppm as a broad signal is typical of intramolecular hydrogen bonding[16].

The $C_2$-OH signal for (II), (IV) and (V) appeared in the region 12.72-13.61 ppm is suggestive of its involvement in intramolecular hydrogen bonding. However, the spectrum for (I) and (III) do not exhibit any signal originating from this $C_2$-hydroxyl group probably indicating the increased stability of intramolecular hydrogen bonding.

Quinone oximes[17] are found to possess *syn-amphi* isomers(Fig.2); similar to nitrosophenol tautomers[18]. Rane et al have suggested that *amphi* form is predominant for quinone oxime derivatives mainly due to the steric effects of C-3 substituents as well as their inductive nature. The signal at~9 ppm is assigned to oximino proton($H_{11}$) which is coupled with $H_9$ in equal intensity suggesting the predominance of amphi form of oxime(Fig. 2). Also, the doublet at~7.9-8.3 ppm for these oxime derivatives supplemented such an observation. $H_{6,7}$ protons appeared as a doublet of doublet at ~7.57-7.79 ppm indicating the spin-spin coupling with $H_5$ and $H_8$ protons, while $H_5$ signal is found to be a doublet. Signal originating from $H_3$ proton for (I) appeared at 6.16 ppm as a singlet same that of lawsone[16].

**Table 3** $^1$H NMR data for lawsone and its oxime derivatives (δ ppm)

| Compound$^+$ | N-OH | $C_2$-OH | $H_3$ | $H_5$ | $H_{6,7}$ | $H_8$ | $-CH_3$ |
|---|---|---|---|---|---|---|---|
| Lawsone | ---- | 11.66(br) | 6.15(s) | 7.90(d) | 7.79(q) | 7.95(d) | ---- |
| (I) | 9.05(d) | ---- | 6.16(s) | 8.00(d) | 7.63(q) | 8.22(d) | ---- |
| (II) | 9.01(d) | 12.72 | ---- | 8.15(d) | 7.57(q) | 8.29(d) | 2.05 |
| (III) | 9.02(d) | ---- | ---- | 8.15(d) | 7.61(q) | 8.30(d) | ---- |
| (IV) | 9.06(d) | 13.61 | ---- | 8.16(d) | 7.66(q) | 8.27(d) | ---- |
| (V) | 9.04(d) | 13.44 | ---- | 8.16(d) | 7.61(q) | 8.28(d) | ---- |

$^+$ ( ) ligand, br : broad, d : doublet, q : quartet, s : singlet

The $^{13}$C NMR data of (I)-(V) is summarized in Table 4. Although, $^{13}$C NMR of quinone oximes has not been fully explored, we have assigned the resonance signals based on the earlier reports on 2-methoxy-1,4-naphthoquinone[19] and lawsone[16].

**Table 4** $^{13}$C NMR data for lawsone and its oxime derivatives(δ ppm)

| Compound$^+$ | $C_1$ | $C_2$ | $C_3$ | $C_4$ | $C_5$ | $C_6$ | $C_7$ | $C_8$ | $C_9$ | $C_{10}$ | $-CH_3$ |
|---|---|---|---|---|---|---|---|---|---|---|---|
| Lawsone | 181.0 | 159.3 | 111.0 | 184.3 | 125.0 | 134.0 | 133.0 | 125.7 | 130.3 | 131.7 | ---- |
| (I) | 156.3 | 143.3 | 109.1 | 178.0 | 122.2 | 131.9 | 131.1 | 126.3 | 130.0 | 131.1 | ---- |
| (II) | 157.0 | 139.0 | 113.1 | 185.0 | 125.7 | 131.7 | 130.6 | 126.1 | 129.2 | 130.1 | 7.9 |
| (III) | 157.8 | 138.8 | 110.8 | 178.1 | 122.4 | 132.4 | 131.7 | 126.5 | 129.4 | 130.2 | ---- |
| (IV) | 159.2 | 138.7 | 111.9 | 177.5 | 124.7 | 131.7 | 129.5 | 125.9 | 128.8 | 129.0 | ---- |
| (V) | 163.0 | 138.5 | 114.0 | 179.5 | 125.4 | 132.1 | 130.0 | 126.8 | 128.9 | 129.1 | ---- |

$^+$ ( ) ligand

It is significantly observed that the $C_1$ signal is substantially shifted towards higher field (by ~18-25 ppm) indicating the influence of basic nitrogen atom of oximino function[20]. Moreover, the adjacent $C_2$ position has also undergone upward shift by ~16-21 ppm. This clearly indicates that $C_2$-hydroxyl group is involved in intramolecular hydrogen bonding leading to the dominance of *amphi*-form of oxime derivatives. The resonating structure of quinonoidal part of naphthoquinones seems to be disturbed due to oximation which is clearly reflected in the increased energy of $C_4$ resonance for (I)-(V) as compared to lawsone. However, other signals originating from remaining carbon atoms are only slightly affected.

### c) Antimicrobial activity of the compounds

The extent of inhibition of *Staphylococcus aureus* by ligands is found to be more pronounced than their complexes[21-24]. However the activity (I), (II) is more inhibitory to *Staphylococcus aureus* as compared to dichlone. (I)-(V) inhibited *Xanthomonas. campestris* significantly than their metal complexes. The extent of inhibition for ligands is more than that of dichlone[25]. The extent of inhibition of *Pseudomonas aeruginosa* by ligands and their complexes is not much altered. However (I), (II), $Er_2$, (III) exhibit increased inhibition against *Pseudomonas aeruginosa*. Erbium complexes in general of (I)-(V); exhibit lesser antimicrobial activity against most of the organisms studied as compared to their oxime derivatives[23,24,26].

**Table 5**. MCC (μg/mL) of ligands and their complexes

| Compound+ | *S.aureus* | *X.campestris* | *P.aeruginosa* | *C.albicans* | *A.niger* |
|---|---|---|---|---|---|
| (I) | 500 | >500 | >500 | >500 | >500 |
| (II) | 500 | >500 | >500 | >500 | >500 |
| (III) | >1000 | >500 | 1000 | 500 | 500 |
| (IV) | >1000 | 1000 | >1000 | 500 | 500 |
| (V) | >1000 | 1000 | >1000 | 500 | 500 |
| $Er_1$ | 500 | 500 | >1000 | 1000 | 500 |
| $Er_2$ | 500 | 500 | 500 | 500 | 500 |
| $Er_3$ | >1000 | 1000 | 1000 | 500 | 500 |
| $Er_4$ | >1000 | 1000 | >1000 | 500 | 500 |
| $Er_5$ | >1000 | 1000 | >1000 | 500 | 500 |
| Dichlone | 200 | 200 | >250 | 50 | 50 |

+( ), ligand; Er, metal complex, Dichlone-standard, DMSO, solvent

**Table 6**. Antimicrobial activity* of the compounds

| Compound+ | *S.aureus* | *X.campestris* | *P.aeruginosa* | *C.albicans* | *A.niger* |
|---|---|---|---|---|---|
| Dichlone | 15 | 08 | 09 | 16 | 08 |
| (I) | 20 | 12 | 11 | 18 | 14 |
| $Er_1$ | 13 | 09 | 10 | 10 | 08 |
| (II) | 22 | 15 | 12 | 22 | 20 |
| $Er_2$ | 14 | 10 | 11 | 12 | 08 |
| (III) | 15 | 15 | 10 | 22 | 25 |
| $Er_3$ | 08 | 09 | 08 | 08 | 08 |
| (IV) | 12 | 10 | 09 | 20 | 21 |
| $Er_4$ | 08 | 08 | 08 | 08 | 08 |
| (V) | 13 | 12 | 09 | 20 | 21 |
| $Er_5$ | 08 | 09 | 08 | 08 | 08 |

*inhibition zone diameters in mm, +( ), ligand; Er, metal complex; DMSO-solvent, Dichlone: standard

Amongst bacteria *Staphylococcus aureus* was more sensitive to (I) and (II). *X. campestris* and *Pseudomonas aeruginosa* are relatively less sensitive which is an indication of their inherent resistance for these compounds. All the compounds, especially the ligands, have better antifungal activity than that of antibacterial as indicated by the highest sensitivity of *Candida albicans* and *Aspergillus niger*[27,28]. It can be concluded that complexes, in general possess lower antimicrobial activity than the ligands. *Staphylococcus aureus* is more sensitive to (I) and (II). The *Candida albicans* and *Aspergillus niger* are highly sensitive to (II), (III), (IV) and (V). The sensitivity of all these fungi is comparable to that of *Staphylococcus aureus*.

**ACKNOWLEDGEMENT**

S.B. Jagtap and N. N. Patil are thankful to UGC, New Delhi for providing Teacher fellowship. The authors thank Department of Chemistry and Department of Microbiology, University of Pune for the facilities provided to carry out this work. Author SBJ is thankful to Dr. V. D. Kelkar, Dr. D.D.Dhavale, Dr. R.S.Kusurkar, Dr. A.S.Kumbhar and Dr. R.C.Chikate for their discussions on characterization of compounds.

**REFERENCES**

1. Williams, R. J. P., *Structure. and Bonding*, **50**,79-119 (1982).
2. Hider, R. C., and Hall, A. D., *Progress in Medicinal Chemistry*, **28**, 41-173 (1991).
3. Laufter, R. B., *Chem. Rev.*, **87**, 901-927 (1987).
4. Yam, V. W. W., Lo, K. K. W., *Coord. Chem. Rev.*, **184**, 157-240 (1999).
5. Gaikwad, S. D., *Ph. D. Thesis*, University of Pune (India), (1997).
6. Dandawate, F. V., Kodolikar, J. G., Kelkar, V. D., Kulkarni, B. A., *Themochim. Acta*, **241**, 103 (1994).
7. Kelkar, V. D., Gokhale, R. R., *Polish J. Chem*, **72**, 655-659 (1998).

8. Jagtap, S. B., Joshi, S. G., Litake, G. M., Ghole, V. S., Kulkarni, B. A., *Met-Based Drugs*, **7**(3), 147-150 (2000).
9. Fieser, L. F., *J. Biol. Chem.*, **133**, 391 (1940).
10. Olsen, W. A., *Aust. J. Hosp. Pharm.*, **9**, 127-133 (1979).
11. Jones, D., Dell, P. A., Sneath, P. H. A., *A Manual of Laboratory Methods*, Academic Press, 35-40 (1984).
12. Ryley, J. F., Rathmell, W. G., *Symposium of British Mycological Society*, 63-87 (1984).
13. Chand, S., Lusunzi , I., Veal, D. A., Williams, L. R., Karuso, P., *J.f Antibiotics*, **47**, 1295-1304 (1994).
14. Pierpont, C. G., Downs H. M., Rukavina, T. G., *J. Am. Chem. Soc.* **96**, 5573 (1974).
15. Padhye, S.B.and Kulkarni, B.A., *J.Magn. Reson.* **16**, 150 (1974).
16. EL-Hendawy, A.M., *Polyhedron*,**10**,No.20/21, 2511-2518(1991).
17. Rane, S. Y., Dhavale, D. D., Muley, M. P., Khan, E. M., *Spectrochim. Acta.* **46A**, 113, (1990).
18. Norris R.K.and Sternhell S., *Aust.J.Chem.*,**22**,935(1969).
19. Hofle G.,*Tetrahedron*,**33**,1963(1977).
20. Buchanan G.W.and Dawwson B.A.,*Can.J.Chem.*,**56**,2200-2204(1978).
21. Lime, O.G. de, Coelho, J. S., Albuquerque, I.L de, Mello, J. F. de, Martin, D. G., Lacerda, A. L., Moraes Souza, M. A. de., *Rev. Inst. Antibiot.* (Recife), **11**, 21-26 (1971).
22. Roushdi, I. M., Ibrahim, E. S., Habib, N. S., *Pharmazie*, **31**, 856-859 (1976).
23. Kelkar, V. D., Rawal, B. M., Kulkarni, P. T., *Indian J. Pharm. Sci.*, **48**, 198-200 (1986)
24. Joshi, C. R., *Indian J. Pharm. Sci.*, **48**, 101 (1986).
25. Tripathi, R. D., Srivastava, H. S., Dixit, S. N., *Experientia*, **34**, 51-52 (1978).
26. Gershon, H., Shanks, L., *Can. J. Microbiol.*, **21**, 1317-1321 (1975).
27. Dandawate, F.V., *Ph.D.*, Thesis, University of Pune, India (1993).
28. Fox, CL, Jr., *Pahlavi Med. J.*, **8**, 45-64 (1977).

**Received: October 30, 2000 – Accepted: November 20, 2000 – Accepted in publishable format: September 10, 2001**

